# Quality of Deaf and Hard-of-Hearing Mobile Apps: Evaluation Using the Mobile App Rating Scale (MARS) With Additional Criteria From a Content Expert

**DOI:** 10.2196/14198

**Published:** 2019-10-30

**Authors:** Ryan Lee Romero, Frederick Kates, Mark Hart, Amanda Ojeda, Itai Meirom, Stephen Hardy

**Affiliations:** 1 College of Public Health and Health Professions University of Florida Gainesville, FL United States

**Keywords:** eHealth, mobile health, mHealth, mobile app, hearing, deaf persons, sign language

## Abstract

**Background:**

The spread of technology and dissemination of knowledge across the World Wide Web has prompted the development of apps for American Sign Language (ASL) translation, interpretation, and syntax recognition. There is limited literature regarding the quality, effectiveness, and appropriateness of mobile health (mHealth) apps for the deaf and hard-of-hearing (DHOH) that pose to aid the DHOH in their everyday communication and activities. Other than the star-rating system with minimal comments regarding quality, the evaluation metrics used to rate mobile apps are commonly subjective.

**Objective:**

This study aimed to evaluate the quality and effectiveness of DHOH apps using a standardized scale. In addition, it also aimed to identify content-specific criteria to improve the evaluation process by using a content expert, and to use the content expert to more accurately evaluate apps and features supporting the DHOH.

**Methods:**

A list of potential apps for evaluation was generated after a preliminary screening for apps related to the DHOH. Inclusion and exclusion criteria were developed to refine the master list of apps. The study modified a standardized rating scale with additional content-specific criteria applicable to the DHOH population for app evaluation. This was accomplished by including a DHOH content expert in the design of content-specific criteria.

**Results:**

The results indicate a clear distinction in Mobile App Rating Scale (MARS) scores among apps within the study’s three app categories: ASL translators (highest score=3.72), speech-to-text (highest score=3.6), and hard-of-hearing assistants (highest score=3.90). Of the 217 apps obtained from the search criteria, 21 apps met the inclusion and exclusion criteria. Furthermore, the limited consideration for measures specific to the target population along with a high app turnover rate suggests opportunities for improved app effectiveness and evaluation.

**Conclusions:**

As more mHealth apps enter the market for the DHOH population, more criteria-based evaluation is needed to ensure the safety and appropriateness of the apps for the intended users. Evaluation of population-specific mHealth apps can benefit from content-specific measurement criteria developed by a content expert in the field.

## Introduction

### Background

The continuous growth of mobile phone technology opens new opportunities for mobile health (mHealth) apps. The improved computing capability, increased memory, and the open operating systems of smartphones support mHealth app development and help shape the future of health care [1,2]. In 2018, there were approximately 205.4 billion mobile apps downloaded worldwide, with a forecasted growth to 258.2 billion by 2022 [3]. This emerging technology can create a more inclusive and accessible environment for the deaf and hard-of-hearing (DHOH) population, representing more than 7 million Americans or over 2% of the US population [4,5]. New mHealth apps leveraging the internet can help to reduce barriers for individuals with disabilities, which is a crucial component of the Americans with Disabilities Act [6]. Disability and health inclusion strategies include identifying and eliminating communication barriers for people with hearing impairments [7]. Universal accessibility of apps that can increase the multidirectional communication between DHOH persons and those who are not is essential for social inclusion [8].

Although there are mHealth apps available to the DHOH population, there is minimal information regarding the quality, features, effectiveness, and maintenance of these apps. Information regarding the status of DHOH apps should be expanded; this information will give consumers valuable knowledge relevant to choosing an app. In addition, information on specific user needs could assist developers in recognizing features desired by the population that have not been fulfilled with the present mobile apps. Relying on star ratings and reviews may be insufficient for app developers and analysts because of the volume of ratings and the usefulness of the information [9]. To evaluate and rank apps relevant to the DHOH in a quantitative manner, a standardized scale is needed, such as the Mobile App Rating Scale (MARS) by Stoyanov et al [10]. The MARS is an evaluation system divided into 5 core sections that can accommodate a wide variety of mHealth apps.

The deaf population can be divided into groups based on different criteria such as the degree of hearing loss, language preference, educational experience, and integration in the Deaf community or the hearing population [11]. Throughout, the study will use DHOH to indicate the DHOH population in the United States. Within this population, there is a distinction to be made between persons identifying as Deaf (uppercase D) and deaf (lowercase D). Those who identify as Deaf are actively engaged in a common Deaf culture and the identity behind the culture and prefer to use American Sign Language (ASL) or use only ASL [12]. Persons identifying as deaf or hard-of-hearing may comprise those who have postlingual hearing loss, prefer to use English over ASL, or choose to associate with the hearing culture [13]. Owing to the unique characteristics of the DHOH population in the United States and the complexity of the ASL [14], there is no commonly used written system for ASL; Web-based text is needed for updating content or simple user queries [15]. In the United States, more than 500,000 individuals use sign language as their primary mode of communication [16]. ASL interpreters are commonly employed to resolve communication barriers between the DHOH and others [17]. The Bureau of Labor Statistics projects 18% growth for the industry from 2016 to 2026, which is much faster than the average for all occupations. The United States is expected to add 12,100 new positions by 2026 [18]. However, it can take years to become fluent in ASL [19], and in many areas of the country, there is a shortage of sign language interpreters [17,20]. Although signing interpreters are a staple of interpersonal communication for the DHOH community, mobile apps can also bolster interpersonal communication in numerous ways [21,22].

### Objective

The aim of this study was to modify a standardized scale to evaluate and rank apps designed for the DHOH. The study sought the use of a content expert to develop content-specific criteria that would gauge the presence of features relevant to the target population.

## Methods

### Search and Selection Criteria

An initial screening of the DHOH apps was conducted in May 2018 across 2 mobile app stores: Apple’s App Store and the Google Play Store (for Android platform). The initial search criteria sought apps aimed toward assisting the DHOH community in the United States.

The following search terms were used: “deaf,” “deaf application,” “deaf hearing,” “hard of hearing,” “sign language translator,” “sign language,” “sign language applications,” “ASL translator,” ASL sign language,” “sign language dictionary,” “sign language keyboard,” “text to speech,” “hearing assistant,” “hearing aid,” “deaf text to speech,” “deaf translator,” and “ASL assistant.”

A list of potential apps to review was generated after a preliminary screening was done based on the app description in the stores and supporting screenshots to establish relevance and initial inclusion. In-store reviews and star ratings were not considered to prevent rater bias. The study was IRB201802567 exempt.

### Inclusion Criteria of Apps and Processes

To best select a list of apps, inclusion and exclusion criteria were developed for the master list of apps with the goals of the study in mind, which were (1) to determine the objective quality of hard-of-hearing apps on the mobile market and (2) to gauge the affinity of these apps to integrate the hard-of-hearing population into the national community via the elimination of a social barrier. As the 2 main resource pools for target apps were the Apple’s App Store and the Google Play Store, only apps from these 2 repositories were considered.

#### Initial Apps Excluded From the Study

**Figure 1:**
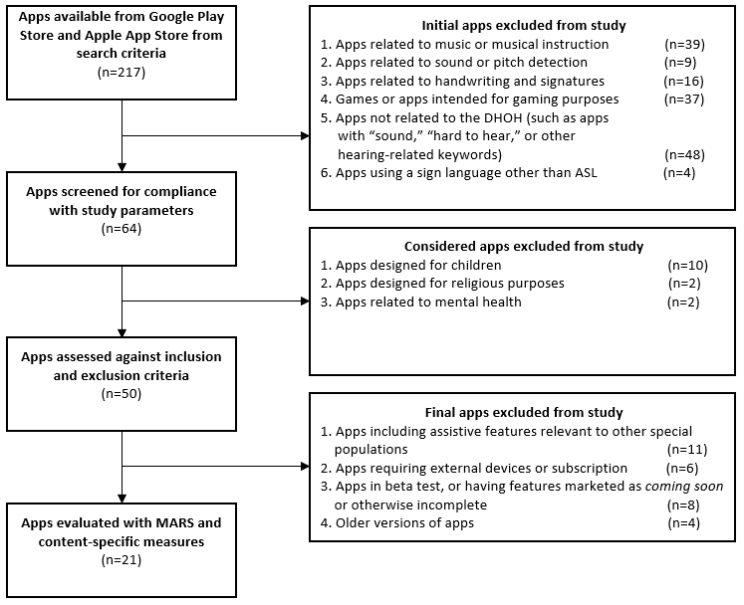
Inclusion and exclusion criteria flowchart. ASL: American Sign Language; DHOH: deaf and hard-of-hearing; MARS: Mobile App Rating Scale.

Apps from both stores (n=217) underwent an initial filtering to obtain a diverse spread of apps related to the DHOH community based on the search criteria. Apps not directly related to the DHOH were not considered further, which include apps related to music or musical instruction (n=39); apps related to sound or pitch detection (n=9); apps related to handwriting or signatures (n=16); games or apps intended for gaming purposes (n=37); apps not otherwise related to the DHOH, or contained keywords related to DHOH (such as apps with “sound,” “hearing,” “hard to hear,” or other hearing-related keywords within the app title) but did not include DHOH features (n=48); and apps using a sign language other than ASL (eg, British Sign Language or French Sign Language; n=4; see Figure 1).

#### Considered Apps Excluded From the Study

After the initial filtering, apps were screened for compatibility with the 3 categories of the study (ASL translators, speech-to-text, and hearing assistants; n=64). Apps having a primary focus other than the aid of the DHOH population were excluded in this step. Apps designed for children (n=10), religious purposes (n=2), or mental health topics (n=2) were not considered for inclusion criteria screening (see Figure 1).

#### Final Apps Excluded From the Study

Remaining apps (n=50) were then assessed against the inclusion and exclusion criteria. Apps meeting 4 final exclusion criteria were not considered for evaluation with the MARS: apps including assistive features relevant to other special populations (apps with assistive features intended for other populations were excluded to standardize the scoring of the study’s content-specific measures; n=11); apps requiring external devices or a subscription for function (n=6); apps in beta test, or having features such as *coming soon* or otherwise incomplete (n=8); and apps representing an older version of an app being considered (n=4; see Figure 1).

The use of mHealth apps typically falls under the definition of assistive technology (AT), which can be a piece of equipment, software program, or product used to increase, maintain, or improve the functional capabilities of persons with disabilities [23]. Therefore, the study includes a content expert (SH) to evaluate the MARS criteria and to create criteria to better match particular assistive apps to specific needs. Owing to the complexity of evaluating ASL apps, particularly for a person who has no hearing difficulties or ASL experience, it was evident that a content expert was needed.

### Apps

**Figure 2:**
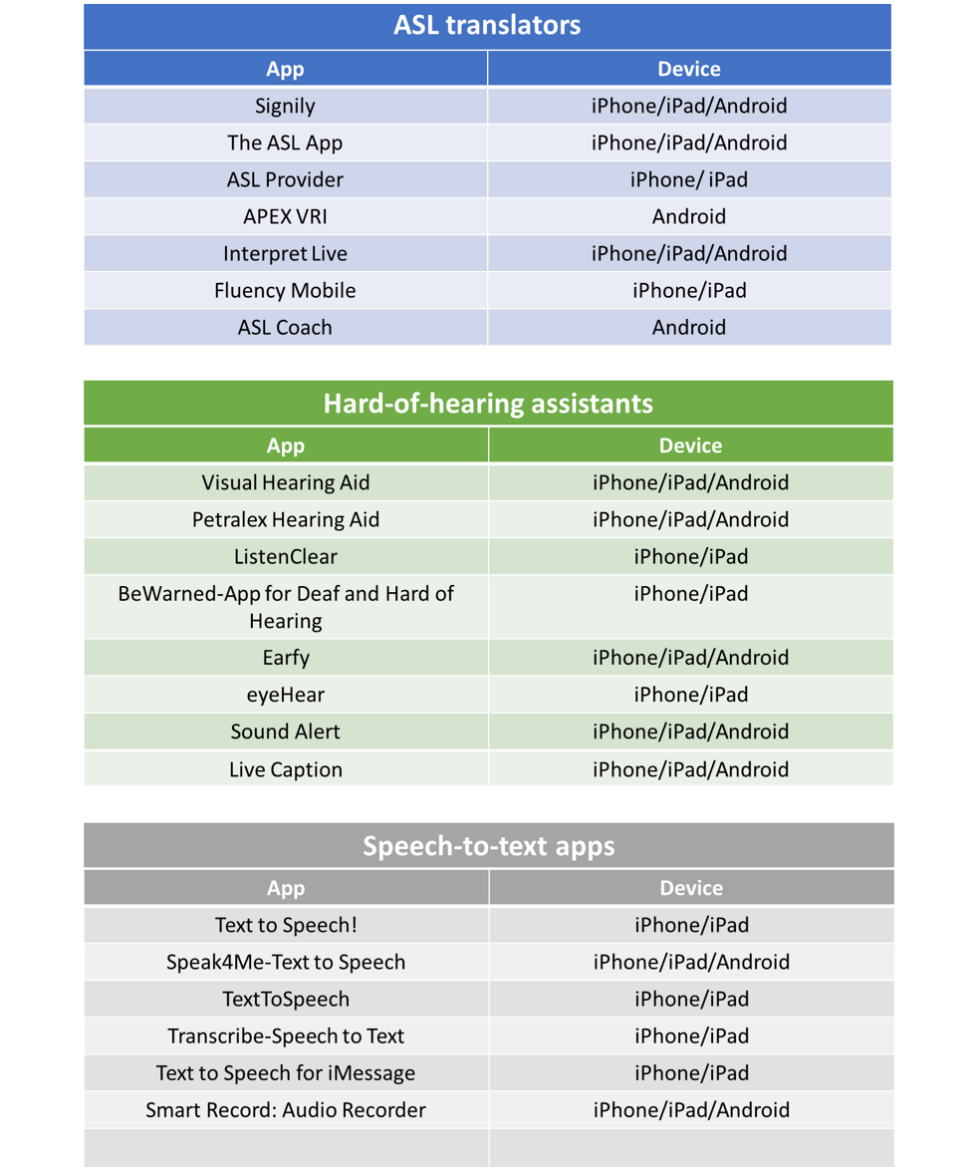
Master list of apps. ASL: American Sign Language.

The development of a multicategory master list of apps allowed for an independent evaluation of each category of apps with the MARS scale and developed content-specific measures (see Figure 2).

#### American Sign Language Translators

Apps providing translational functionality to and from ASL were considered in this section. Statistically, “congenital hearing loss affects two to three infants per 1,000 live births” [24]. As ASL is the main component of communication for many Deaf persons in the United States [25], in addition to the unique accessibility needs of Deaf persons regarding communication [11], it was appropriate to allocate an entire section of study to this app category. ASL is unique from verbal language as it provides a mode of communication based on symbols and visual cues [25]. In addition, those who are prelingually deafened (and who typically associate with the Deaf culture) are more reliant on ASL-based communication than verbal or written English [26]. Apps that serve to translate sign language to and from words, whether it be by visual interpretation or ASL dictionary function, were sorted into this category.

#### Speech-to-Text

Threaded speech-to-text apps are also helping individuals with hearing loss to take part in conversations with family and groups of people. The best hard-of-hearing lip-readers can understand approximately 30% of a dialogue [27]. With threaded speech-to-text apps, the accuracy of conversation increases to around 80% to 90% [28]. Speech-to-text allows for a more conversational-speed communication between the hard-of-hearing and others. In the current app market, there are a substantial number of these apps competing to be used, yet many are not geared toward the hard-of-hearing population. Although this category of apps poses great benefit to those who were postlingually deafened or are hard of hearing, speech-to-text translators may not be particularly useful toward individuals who were prelingually deafened, stemming from a lower level of English usage and comprehension [29].

#### Hearing Assistants

The study’s third category included apps that sought to improve user communication and interaction in everyday life. This section was created to give the study the opportunity to analyze apps that did not fit into the other 2 categories yet provided some feature that positively affected a DHOH person’s ability to communicate. A content expert was used to determine whether or not an app provided some benefit to a DHOH person in social function or public navigation.

### Data Collection and the Mobile App Rating Scale

Decision was made to use a reliable and flexible app quality rating scale designed by expert research panelists to assess the quality of mHealth apps via multiple descriptive factors [10]. The MARS was developed to satisfy the need for a reliable and objective scale that could rate the degree to which mHealth apps satisfy the defined quality criteria [10]. The study used the 4 MARS classifications, plus an additional *content-specific* section: engagement, functionality, aesthetics, and information quality. The subjective quality section of the MARS was substituted for the custom-designed content-specific measures section to better gauge app quality and appropriateness. With the inclusion of the study’s 4 custom-designed criteria, apps were evaluated on 23 metrics. All the metrics were quantified by assigning integer values: 1=poor, 2=fair, 3=acceptable, 4=good, and 5=excellent.

The mobile apps using the MARS were analyzed by 3 members of the research team (RR, IM, and AO), designated as *raters*. Each rater underwent 2 training sessions to correctly attribute scores from the MARS to the apps. These training sessions were supplemented by a video developed by the MARS creators to aid in the rater training and calibration [30]. The MARS training video is a reference created by the authors of the MARS to explain the purpose of the scale, the app characteristics that each subsection of the scale measures, and the guidelines for evaluating each subsection. The video describes the relative quality of each app characteristic that is appropriate for each score (poor-excellent), along with examples of quality features. Furthermore, the MARS training video acted as a knowledge base during the construction of content-specific measures and generated ideas as to how content-specific criteria may be scored. These ideas aided the refinement of the content-specific questions after development with the content expert. The 3 raters are members of the College of Public Health who are involved in hard-of-hearing research. The raters were all trained with knowledge from the content expert to give a shared and equal understanding of the needs and characteristics of the DHOH population. A total of 3 control apps were used to determine the intraclass correlation coefficients among raters based on Shrout and Fleiss’ guidelines [31]. A test similar to Fleiss’ kappa, the Krippendorff alpha, was used to test for interrater agreement [32]. An alpha of .91 was obtained. All issues regarding agreeability were discussed among team members and reevaluated for appropriate concurrence.

### Use of a Content Expert and “Content-Specific” Measures

Content experts can be useful when determining the relevance of a proposed study to a specific population or subject [33]. Specifically, the study revolves around the needs, desires, and attributes of the DHOH population. The study’s content expert (SH) is a professor of ASL who himself is deaf. The Deaf culture is a broad network of beliefs, values, and rules for behaviors that are incorporated into the lives of many with hearing difficulties [12]. The use of a content expert allowed the study design to consider the needs and desires of this population as related to mobile app features so that valid and meaningful metrics could be designed to test for the presence of such features.

An additional scoring section, the content-specific section, was tailored with a content expert (SH) for hearing-specific apps following the MARS authors’ interest in criteria applicable to specific populations [10]. This section included 4 subfactors: signing space, distractions, assistive features, and societal integration potential. To help create the content-specific classifications and improve validity, a content expert with experience in teaching ASL and ASL linguistics and Deaf studies was added to the study. After the initial review of apps, concerns were discussed with the content expert to further refine the criteria. All the category factors were quantified by assigning integer values: 1=poor, 2=fair, 3=acceptable, 4=good, and 5=excellent.

#### Signing Space

Evaluating the signing space is an important measurement criterion as this area gauges the digital interpreter’s use of sign language. In sign language, the signing space encompasses the distinctive locations surrounding the signer [34], particularly the space in front of the signer extending from the waist to the forehead [35]. The content expert emphasized that signing in apps should be in a consistent space and easy to follow. The content expert developed several questions with measurable criteria to assist the evaluators in scoring apps: Does the signing stay within approximately 12 inches of the body’s center mass? Does the reader have to concentrate on entirely different areas when facial expressions are used? Is the signing too fast or too slow?

#### Distractions

Distraction evaluation includes any attribute or behavior by the app or interpreter that may be distracting to the reader. These include distracting clothing (with patterns or multiple colors), bright nail polish or jewelry, and extraneous events occurring in the background or the background color [15,36]. Furthermore, signers should consider the contrast between their skin complexion and their clothing and the background to focus the attention around the hands [37].

#### Assistive Features

The initial purpose of hard-of-hearing apps and translators is to allow those with hearing difficulties to achieve social function similar to those who are not hard of hearing. One way the apps can achieve this is by integrating the assistive features within their menus and the graphical user interfaces (GUIs). These assistive features can be (but are not limited to) enlarged buttons and text, a magnifier function, closed captioning on app dialogue, and slow scrolling text of a large font style.

#### Societal Integration

One desirable function of apps that seek to assist the DHOH and similar populations would be to provide functions that further aid in the user’s inclusion and experience in society. The nature of certain apps may lead them to have a higher affinity for this criterion, but overall, this section can be rated by reviewing the potential for a particular app to integrate a DHOH person into society (via social outings, dating, or simply acting as an aid in public).

## Results

### App Turnover

One outcome not expected was the high level of *app turnover* noted in the ASL translator section. For the study’s purposes, *app turnover* has been defined as an app being added to the master list of apps for evaluation but later being wholly inaccessible or unusable because of its removal from software repositories, extremely poor design, or abandonment by the developer. A total of 5 of the 7 apps were lost to app turnover: ASL Provider, APEX VRI, Interpret Live, Fluency Mobile, and ASL Coach. Although the study was left with a reasonable distribution of apps to evaluate, it should be noted that the high level of app turnover serves as a general reflection of the state of hard-of-hearing apps.

### Scoring for American Sign Language Translators

Upon completion of scoring for the ASL translator apps, the consensus among the 3 evaluators was that this category of apps had quality apps, yet it was subject to a large amount of *app turnover*, as previously discussed. Of the 7 ASL translator apps selected for evaluation, only 2 were accessible or even discoverable in either app repositories. The best app rated in the category was Signily (3.72/5), with the second best being The ASL App (3.642/5). Both apps had acceptable to good general attributes and acceptable content-specific qualities (see Table 1).

### Scoring for Speech-to-Text

Of the 6 apps tested in the speech-to-text category, all 6 apps were still accessible at the end of the scoring period. The best app in the category was Text to Speech! by Gwyn Durbridge (3.595/5), followed by Speak4Me–Text to Speech (3.584/5) and Smart Record: Audio Recorder (3.48/5). This category of apps had the lowest average score for all 4 general attribute categories, and the lowest average score in the content-specific measures section (see Table 1). However, because these apps were not explicitly designed for the DHOH population, they should only be faulted if drastic improvements toward the target population’s needs would be easily achievable.

### Scoring for Hearing Assistants

Of the 8 apps tested in the hearing assistant category, all 8 apps were still accessible at the end of the scoring period. The best app in the hearing assistant category was ListenClear (3.90/5), followed by Petralex Hearing Aid (3.89/5) and Sound Alert (3.73). Of the 3 categories evaluated, consensus was reached that this particular category boasted the most consistent level of engagement across apps (see Table 1). In addition, the average functionality score was the highest in this category. Although the quality of the general attributes of these apps is either acceptable or good, several of the apps had only fair or poor scores for the content-specific measures (see Table 1). There was a notable variance among raters in the content-specific section while evaluating the app *ListenClear*. Although the ListenClear app has an exceptional GUI, assistive features, and minimal distractions, it would be desirable for this app to have increased societal integration features considering how it is marketed to the DHOH population (as a hearing assistant).

**Table 1:** Mobile App Rating Scale app quality ratings.

Mobile health app ranking		Engagement	Functionality	Aesthetics	Information quality	Content-specific criteria^a^
**ASL^b^** **translators**						
	Signily	3.8	3.8	3.2	3.8	3.9
	The ASL App	3.0	4.4	3.9	3.8	3.0
**DHOH^c^** **assistants**						
	ListenClear	4.1	4.3	4.7	4.0	2.5
	Petralex Hearing Aid	3.9	4.3	3.9	3.9	3.4
	Sound Alert	3.8	4.3	4.1	3.5	3.0
	BeWarned–App for DHOH	4.2	4.1	3.7	3.6	3.1
	Visual Hearing Aid	3.3	4.1	3.2	3.6	2.0
	eyeHear	2.7	3.8	3.0	3.1	2.2
	Live Caption	2.8	3.8	2.6	2.9	2.4
	Earfy	2.9	3.3	2.0	2.4	1.8
**Speech-to-text**						
	Text to Speech!	3.9	3.8	3.6	3.5	3.2
	Speak4Me–Text to Speech	3.9	4.1	3.4	3.4	3.0
	Smart Record: Audio Recorder	3.1	3.8	4.1	4.4	1.0
	Transcribe–Speech to Text	3.1	3.3	3.4	3.2	2.1
	TextToSpeech (Iconic Solutions, LLC)	2.6	2.8	2.7	2.4	1.5
	Text to Speech for iMessage	1.9	1.7	1.9	2.1	1.3

^a^Content-specific criteria are based on the interest in an *app-specific* section by the original Mobile App Rating Scale authors to evaluate apps for specific populations.

^b^ASL: American Sign Language.

^c^DHOH: deaf and hard-of-hearing.

## Discussion

### The State of Hard-of-Hearing Assistance Technology

There is both a need and demand for continuing development of Android and iOS app support for the DHOH. This study’s findings agree with previous findings that app developers prefer Android and iOS for their projects [38]. Of the 217 apps from the search criteria, 50 apps were assessed against the inclusion and exclusion criteria for the study. Given the relatively low yield of candidate apps from the App Store and Google Play Store, it is thought necessary for more development of apps targeted toward the DHOH population. A refinement of the master list of apps to 21 apps based on the inclusion and exclusion criteria was thought sufficient to mitigate any anomalies in the study. When it came time to evaluate the apps, however, it was observed that 5 of the 7 hard-of-hearing assistants had become unavailable or had otherwise disappeared from the app stores. This suggests a high rate of app turnover in the subject field, which may contribute to the limited availability of quality DHOH apps. It may be inferred that DHOH apps are experiencing high turnover rates, consistent with the current AT turnover rates as high as 75% to 80% [39,40]. It seems apparent that although these apps may have been designed by skilled developers, features that may be basic necessities to some persons in the DHOH population are being overlooked or poorly implemented. Furthermore, the relative lack of high-level features, such as an app’s ability to act as a social advocate for the user, underlines the state of the DHOH apps: available, but limited in scope.

### The Value of Hard-of-Hearing Assistance Technology

One of the tenets of the study was to determine the quality of the available DHOH apps so that it may be inferred on their value to DHOH persons. Given the current state of mobile assistance technology for the intended population, it is reasonable to say that these apps provide augmentation to a deaf or hard-of-hearing person’s ability to navigate in public, interact both publicly and with family, and be connected to other deaf persons through the availability of features that facilitate interpersonal interaction. It is notable, however, that there should be more research into other purposes for these apps. For example, there may be a useful purpose for these kinds of apps in both domestic and emergency scenarios such as hurricanes or severe storms. Indeed, the development of emergency AT could help to reduce the disproportionately high level of morbidity among the DHOH during natural disasters [41]. Mobile apps or devices could have a preprogrammed functionality that assists DHOH persons in an emergency, although this warrants further research.

### Use of a Content Expert to Evaluate Mobile Apps

One measure seeking to validate the study’s design and metrics was the consultation of a content expert (SH) who could act as a representative of the DHOH community with the intent of gauging the quality and applicability of DHOH apps. As a deaf individual, he was able to advocate for the needs of the target population from a position of membership; the study gained insight into the needs of the population along with reasonable knowledge to design content-specific measures included in the MARS. This proved invaluable in guiding the direction of the study to focus not only on the features of the apps that would be representative of a quality app but also on those that could augment the interpersonal and social capability of the user.

### Strengths and Limitations

The study leverages the MARS, which is considered a valid and reliable scale for evaluating mHealth apps. The study introduces a novel MARS modification to create content-specific criteria using a content expert to address the needs of intended end users who will use the apps. One of the limitations of this review was that the search was limited to US app stores. This limitation might have restricted the results and the quality of the apps for the DHOH, particularly if other countries are further ahead in the development of DHOH apps. Only DHOH apps that are publicly available were included, which could have excluded apps developed by a specific health care network that focuses on the DHOH population. A relatively small sample of apps was included in the study. The entire pool of apps for the DHOH was not large to begin with, which may be representative of the DHOH population being a smaller segment of the entire population. There was a high rate of DHOH app turnover, which might be due to the information quality, or that the app is not meeting the needs of the DHOH population**.**

### Future Research

The authors of this study suggest continuing research into the use of the modified MARS scale with content-specific (app-specific) criteria developed with content experts to evaluate mHealth apps for different populations. Further research into the use of content experts while designing a study, and their effect on the validity of subject-specific content will elucidate more information about the potential benefits of using content experts to design content-specific metrics. Additional research is needed for both mHealth apps and DHOH apps to establish additional criteria to measure information quality in terms of patient safety and privacy. Being able to measure the risk of patient safety is a growing concern as some mHealth apps do not follow evidence-based guidelines, or the app developers have little or no medical training [42,43]. Other research opportunities would be to design some app evaluation criteria that can aggregate different types of apps with some beneficial DHOH features (not directly intended for DHOH users). For example, in this study, the initial search terms identified apps such as audio analyzers and sound detectors, which were excluded as they did not have DHOH features. However, 1 sound detection app, eezySoundDetector, recognized the potential to assist the DHOH by providing flashing lights or vibrating alarms for a fire or a crying child. There were other types of apps that were excluded for only having minimal DHOH features, yet these might make a difference for the DHOH in a predominantly hearing world. Developing an evaluation tool that can aggregate a wider variety of apps with different assistive features could be a significant contribution to the development of mHealth apps. In addition, adding a qualitative component to studies that survey user preferences and feedback on the usability of specific mHealth apps has shown to be effective. A preliminary study of a sound detection algorithm that used a training and feedback survey from the DHOH got firsthand insights about detection preferences, such as running water in the home or a printer in the work environment, which improve app usability [44]. Finally, this study can be expanded with a follow-up review on the highest ranked DHOH apps to access effectiveness and tangible gains identified by the users, as well as to measure the DHOH app turnover.

### Conclusions

The MARS remains a high-fidelity tool for app evaluation. The study emphasizes the value of including a content expert early in the mHealth app development process as well as the evaluation process to improve effectiveness and to assist in making criteria-based recommendations to end users. The focus on mHealth apps for the DHOH population illustrates the importance of including a related content expert. For someone who has a hearing difficulty, it can be both reassuring and empowering to see mHealth app developers, evaluators, and providers, who recommend these products, value the understanding of the needs of the intended users. This can be particularly true for an individual with hearing difficulties who struggles with his or her identity in a world set in an overwhelmingly hearing context.

## Abbreviations

ASL: American Sign Language
AT: assistive technology
DHOH: deaf and hard-of-hearing
GUI: graphical user interface
MARS: Mobile App Rating Scale
mHealth: mobile health
